# The natural history of sound localization in mammals – a story of neuronal inhibition

**DOI:** 10.3389/fncir.2014.00116

**Published:** 2014-10-01

**Authors:** Benedikt Grothe, Michael Pecka

**Affiliations:** Division of Neurobiology, Department of Biology II, Ludwig Maximilians University MunichMunich, Germany

**Keywords:** MSO, LSO, evolution, glycine, GABA, archosaurs, birds, binaural hearing

## Abstract

Our concepts of sound localization in the vertebrate brain are widely based on the general assumption that both the ability to detect air-borne sounds and the neuronal processing are homologous in archosaurs (present day crocodiles and birds) and mammals. Yet studies repeatedly report conflicting results on the neuronal circuits and mechanisms, in particular the role of inhibition, as well as the coding strategies between avian and mammalian model systems. Here we argue that mammalian and avian phylogeny of spatial hearing is characterized by a convergent evolution of hearing air-borne sounds rather than by homology. In particular, the different evolutionary origins of tympanic ears and the different availability of binaural cues in early mammals and archosaurs imposed distinct constraints on the respective binaural processing mechanisms. The role of synaptic inhibition in generating binaural spatial sensitivity in mammals is highlighted, as it reveals a unifying principle of mammalian circuit design for encoding sound position. Together, we combine evolutionary, anatomical and physiological arguments for making a clear distinction between mammalian processing mechanisms and coding strategies and those of archosaurs. We emphasize that a consideration of the convergent nature of neuronal mechanisms will significantly increase the explanatory power of studies of spatial processing in both mammals and birds.

## THE EVOLUTIONARY ORIGIN OF HEARING

It has been established for all groups of gnathostomes (jawed vertebrates) that hearing via secondary receptor types, namely hair-cells in the inner ear is directly related to or derived from the same primary substrate – vestibular sensory epithelia (Fritzsch et al., 2002). This strongly supports an early date for the primordial origin of hearing in vertebrates in relation to the encoding of substrate sounds or sounds conducted via bones (e.g., the jaws, jaw joint and the joint-supporting structure, the hyomandibular bone, Manley, 1973). A common origin of substrate hearing is also supported by the overall similarity between the auditory pathways in different vertebrate groups and their close kinship to the similarly hair-cell-driven lateral-line system pathway found in fish. This assertion is amply supported by molecular and developmental studies that underline the overall similarity between these systems, implying that all hearing originated with the detection of aquatic particle motion or substrate sound by mechanical stimulation of vestibular (or lateral-line) hair-cells (Striedter, 1991; Fritzsch et al., 2002; Manley et al., 2004). Therefore, the hair-cell-based reception of non-air-born sound can be considered as basically homologous across all jawed vertebrates.

**FIGURE 1:**
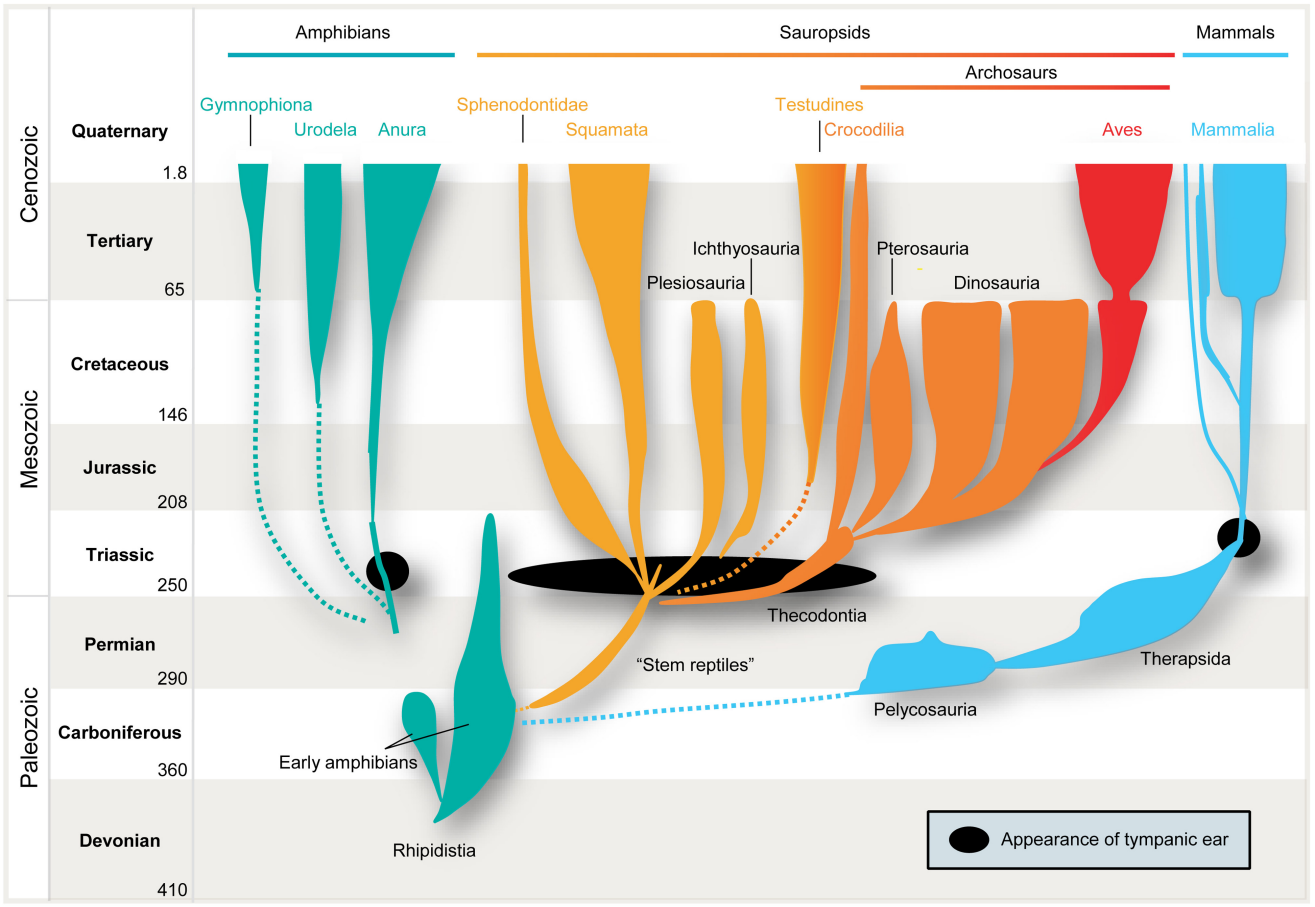
**Parallel evolution of vertebrate ears.** Tympanic middle-ears capable of receiving air-borne sound evolved separately among the ancestors of mammals (blue), modern frogs (“Anura,” green), “reptiles” (yellow), and birds (“Archosaurs,” orange/red) in the Triassic ∼210–230 million years ago (indicated by black closed circles/oval). Note that no common ancestor with tympanic ears had existed.

However, the issue becomes much more complex when we consider the localization of air-borne sounds. Here the concept of general homology is of no help, simply because several prerequisites have to be taken into account. In particular, efficient detection of air-borne sound requires impedance-matching devices (e.g., middle-ear bones, because jaw bones are too big to vibrate in response to air-borne sounds) and imposes specific evolutionary constraints on all neuronal structures and subsequent encoding strategies. Only in very small animals, e.g., some minute frogs, can bony elements that lack a tympanum be mechanically stimulated by air-borne sounds, and thereby directly activate the inner ear (Boistel et al., 2013). Early amniotes were not as diminutive as that. Hence, their bones were too large and massive to be displaced by air-borne pressure waves. Consequently, tympani and specialized middle-ears evolved to detect air-borne sounds. Moreover, these structures developed several times independently, namely in frogs (or some of their ancestors), sauropsids (reptiles and birds), and in mammals (Allin, 1975; Clack, 1997; **Figure 1**). In all these lineages, middle-ears derived from the same precursors, namely from the paired structure that supported the jaw joints: solely from the hyomandibular bone in non-mammals, and from three bones in mammals, specifically the “primary” jaw joint comprising the articular, quadratum, and hyomandibular bones (Reichert, 1837; Gaupp, 1913). These bones originally served both as jaws and to transmit sounds from the jaw via the hyomandibular bone, which supported the jaw joint at the otic region of the skull, by means of bone conduction. At least for mammals, this evolutionary pathway is clearly evidenced in the fossil record. Nevertheless, there is ongoing debate over how often tympanic ears might have evolved independently within the sauropsids (Clack, 2002). Moreover, some authors suggest an independent origin in monotremes and therian mammals (Rich et al., 2005) – a contention which is disputed by others (Rowe et al., 2008). In any case, evolution of the tympanic ear for transmission of air-borne sounds did not follow a single trajectory from a common origin, but represents a classic example of parallel evolution in response to a common selection pressure.

It is therefore safe to assume as a basis for this review that the mammalian tympanic ear evolved independently of those found in all other tetrapods – most importantly in this context, the archosaurs (crocodiles, pterosaurs, dinosaurs and their descendants, birds; **Figure 1** presents a simplified scenario). This picture is supported by the fossil record which confirms that the relevant ancestors (for instance, the predecessors of pelycosaurs, therapsids, and mammals) did not have tympanic ears (Hotton, 1959; Hopson, 1966; Clack, 1997). Further confirmation is provided by comparative anatomy, classical embryology (Rodríguez-Vázquez, 2005) and, more recently, evidence from comparative gene expression studies (reviewed by Sienknecht, 2013) and the role of the neural crest in inserting the bones into the middle-ear cavity (Thompson and Tucker, 2013).

Given that hearing of air-borne sounds evolved more than once, we have to take its evolutionary starting point and the subsequent phylogenetic events into account if we wish to reconstruct the evolution of binaural hearing in different lineages. This approach will also help us to understand and appreciate differences in the structures and processing strategies utilized for this purpose by birds and mammals. Importantly, a precise concept of homology is essential, and we therefore will employ the term “homology” only where it is clear that the structures or functions in question (like specific groups of neurons or pathways) have a common developmental origin and served the same function in the last common ancestor. Otherwise we use the term “analogy.” Note that this distinction does not call into question the overall homology of hair-cell based sound reception *per se*, as mentioned above.

## THE ORIGINS OF SPATIAL HEARING

Binaural sound localization circuits have developed in the context of the processing of air-borne sounds – be it for the purpose of localizing sources, segregating concurrent sounds, or distinguishing primary sounds from echoes. Their development does not exclude the possible use of common ancestral circuits, albeit not specialized for processing binaural cues (see below).

In this context we need to consider the types of spatial acoustic cues to which a given group of animals that developed middle-ears would have been exposed in significant magnitude. This issue relates directly to the anatomy of the skull and the tympanic ear itself. Thus, we first have to take into account what animals were like when they “invented” middle-ears.

We first briefly turn to the three classes of acoustic cues that animals can theoretically use (for a more detailed description see Grothe et al., 2010). First there are *spectral cues* that change when a sound-source moves from one position in space to another. Such changes are most prominent when a sound moves in the vertical plane and thus thought of as monaural. Particularly in animals with prominent outer ears (pinnae), long ear canals and well-developed high-frequency hearing – i.e., most mammals – the complex reflection patterns created by the pinna and ear canal can lead to frequency-specific amplifications, attenuations and even cancelations (defined as so-called “head-related transfer functions,” HRTFs). These effects, however, are not fixed but depend on the direction from which the incoming sounds impinge on the pinna and ear canal. Moreover, the shape and size of the head, and even body posture, can modulate such effects (Blauert, 1997).

In mammals, spectral cues are used for localizing sounds in the vertical plane, where they change most. Not much is known about the use of spectral cues in non-mammalian vertebrates, but because of the nature of their skulls, such signals are unlikely to play a prominent role in reptile and bird sound localization. Furthermore, spectral cues are particularly pronounced at higher frequencies, which most reptiles and birds cannot hear (see below). However, they are of particular relevance to mammals, especially early in their evolution (see below).

**FIGURE 2:**
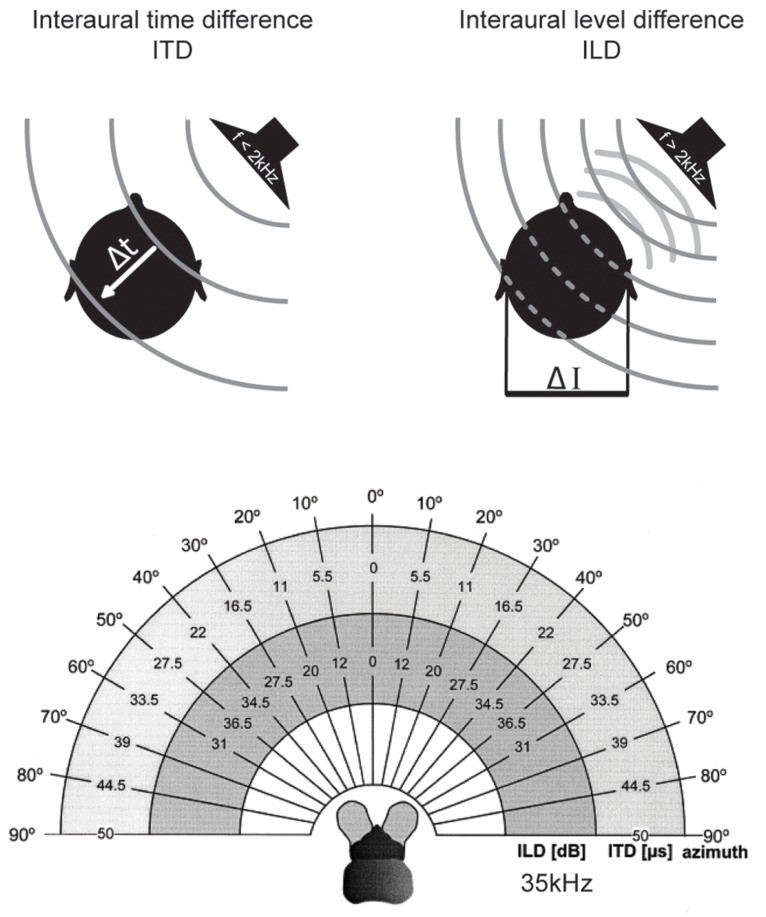
**Binaural cues for sound localization depend on sound frequency and head size. Upper left**: interaural time differences (ITDs): for frequencies below ∼2 kHz, the difference in the arrival time (Δt) of a sound wave (gray lines) at the two ears is used to localize a sound-source in the horizontal plane. ITDs depend on the angle of the sound-source relative to the head axis and the interaural distance (i.e., head size) of the individual. **Upper right**: interaural level differences (ILDs): for frequencies higher than ∼2 kHz, the shadowing effect of the head creates differences in the intensity of the sounds at the two ears (ΔI) that are utilized for sound localization in the horizontal plane. ILDs for a particular sound-source position increase with increasing frequencies. **Lower**: range of ILDs (inner hemicycle) and ITDs (middle hemicycle) are illustrated across the range of azimuthal sound-source positions (outer hemicycle) for a small mammal (the bat *Molussus ater*). While ITDs are minute even for the most lateralized sound-source positions (±50 μs), sizable ILDs are generated by the relatively small head already at moderately high frequencies (35 kHz for this example). Modified with permission from Harnischfeger et al. (1985).

The most important cues for localizing sounds in the horizontal plane are the two binaural cues, interaural time and level differences (Rayleigh, 1907) which depend on frequency and head features (Erulkar, 1972). Interaural time differences (ITDs) – the difference in the time-of-arrival of a sound at the two ears – occurs when the sound-source is not equidistant from both ears. ITDs increase with increasing lateral displacement from the sagittal plane, i.e., to the left or right. Maximal ITDs occur when a sound comes from 90° to the left or right (**Figure 2**). Since in most animals the maximal durations of ITDs are far down in the sub-millisecond range (compare this to the average duration of action potentials of about 1 ms), ITD processing requires either dedicated anatomical specializations – including acoustic/mechanical interferences [as in some insects (Michelsen, 1994) but also in frogs and to some degree in sauropsids (Christensen-Dalsgaard, 2011)]– and/or very specific neuronal adaptations at the level of nerve-cell membranes, synapses, axons, and entire circuits (for review: Grothe et al., 2010).

Finally, the head has a shadowing effect on sound coming from off the sagittal plane. For instance, a sound originating in the horizontal plane but 90° to the left will reach the right ear only after having been attenuated by the head, which lies between the contralateral ear and the sound-source. This shadowing effect results in an interaural level difference (ILD) between the sounds reaching the two ears (**Figure 2**). ILDs are frequency dependent, with high frequencies being affected most and low frequencies being almost unaffected (Rayleigh, 1907) – at least for sounds in the far field (note that in the near range, ILDs can occur even for lower frequencies; Shinn-Cunningham et al., 2000). Head width determines the frequency at which ILDs become relevant (as a rule of thumb: if the wavelength is shorter than the head width, significant ILDs will occur; for humans this corresponds to frequencies >1.3 kHz). Therefore, even very small animals can experience large ILDs at high frequencies (**Figure 2**; Erulkar, 1972; Harnischfeger et al., 1985). On the other hand, even large animals cannot exploit ILDs if they can hear only low frequencies. The former group does not need to process ITDs of only a few 10s of μs, since they can avail of the ILD information. In contrast, the latter have to use ITDs – albeit ITDs that extend to a few 100 μs, and thus these ITDs will have been potentially capable of affecting the response properties of auditory neurons when these systems evolved (Grothe, 2000).

Hence, which cue an animal uses depends on its head size as well as its hearing range. Given that features of the head differed among the early tetrapods that developed tympanic ears (see below), it would not be surprising if different structural and circuit adaptations were to develop in different lineages. Moreover, different evolutionary starting points, in terms of which binaural cue was used first (and why), inevitably should have impacted on the neuronal coding strategy in a given group of animals. This is why understanding spatial hearing depends on taking the evolutionary history of hearing into account.

For reasons that are not yet understood, tympanic ears in tetrapods appeared during an – evolutionarily speaking – rather short time span of 10, maybe 20 million years in the Triassic Period (Clack, 1997). This was only some 150 million years after their amphibian ancestors had first moved onto land and probably only 100 million years after our lineage (the synapsid line leading to Pelycosauria, then Therapsida and finally Mammalia; **Figure 1**) diverged from all other land vertebrates, and apparently also after the Diapsida (most reptiles including archosaurs) had split into several subgroups (see **Figure 1**). The various groups that independently developed tympanic ears in the Triassic were very different, both in terms of anatomy and lifestyle (Tucker and Benton, 1982; Golonka, 2007; Ezcurra, 2010). Moreover, the anatomy of the middle-ear differed significantly between these groups (Manley, 2010). This difference is highly significant for the understanding of the evolution of air-born hearing, because middle-ear anatomy not only is crucial for matching the difference in sound impedance between the outer air and the fluid in the inner ear, but also defines the frequency range transmitted to the sensory epithelium. Frogs and all sauropsids only use one middle-ear bone whose size, mass and mechanics favor low-frequency conduction (see below). In contrast, the mammalian middle-ear evolved right from the start as a very small, low-mass, three-boned structure that favored higher frequency sound conduction. This has significant consequences for their original hearing range and, hence, for the “starting point” at which the evolution of their detection systems for air-borne sounds began. The original hearing range, in turn, had a major impact on the “choice” of binaural cue to be utilized for sound localization. This should be reflected in the neurobiology of hearing of recent animals. We now consider the different combinations of anatomical factors that would be expected to favor the exploitation of a specific spatial cue.

(1) The mechanics of the early middle-ears determined the frequency range of air-borne sounds that could be detected (Fleischer, 1978; Rosowski et al., 1999; Huang et al., 2000). Middle-ear systems that preferentially transmit low-frequency sounds would suggest high selection pressures for ITDs, simply because ILDs are not relevant in that context. In contrast, middle-ears that can transmit high frequencies would favor ILD processing systems (Erulkar, 1972; see above).
(2) The overall size of the animals that first acquired the capacity to use air-borne sound and, hence, that of their skulls, is particularly relevant for assessing how likely or unlikely it is that ITDs were used. Larger heads normally imply larger interaural distances and hence longer ITDs (Kuhn, 1977; Brown and May, 2005). This is relevant since ITD processing involves microsecond precision, which is rather unusual given that action potential duration (on average 1 ms), synaptic delays (>0.5 ms) and jitter (± 100s of μs) significantly compromise the acuity required, as circuits built of neurons with highly specialized membrane properties, synapses and axonal features had not yet evolved. Therefore, it seems plausible to assume that animals developed ITD coding only if ILDs were not significant and if the dimensions of their skulls allowed for ITDs that were long enough to be registered by some neurons in the early auditory pathways (>several 100s of μs). This, of course, does not preclude the evolution of ITD processing in small mammals (reviewed in Grothe, 2000; Köppl, 2009; Manley, 2010) that need to process low frequencies (and such mammals will be discussed later). It is rather a question of likelihood and feasibility.
(3) It also matters whether the original tympanic ears functioned as isolated pressure receivers or whether the two middle-ear cavities were acoustically connected and functioned as a pressure-gradient receiver (Christensen-Dalsgaard, 2005; Köppl, 2009; Manley, 2010). In the former case, the maximal ITD is roughly defined by the absolute interaural distance (i.e., the width of the skull). In the latter, interferences between sounds propagating from one middle-ear cavity to the other can magnify ITDs (Hyson et al., 1994) which helps small animals that strongly rely in low-frequency hearing to gain at least some spatial information from ITDs.
(4) In addition, acoustic and social context, e.g., acoustic communication, either as evolutionary driving force or simply as a co-evolved feature, may have been relevant (Endler, 1993). Larger animals, particularly animals with a larynx (like mammals), are likely to produce call signals at lower frequencies, owing to the resonance frequencies of their sound-producing systems. Although secondary by definition (they can only develop after hearing has been established), such contextual elements can function as an evolutionary feedback system, potent driving force, and selection pressure.

Taking all of these factors into account and combining them with knowledge available from comparative neuroanatomy and physiology, we draw a number of plausible inferences as to how a given group was equipped for the development of spatial hearing and how the initial system evolved further within the group itself. The general concept herein is the following (see also **Figure 9**): physical configurations (head size and hearing range) during the time of the middle-ear development determine the cue that can most easily be exploited for sound localization for a given taxon. The binaural cue in turn shapes the emergence of distinct neuronal mechanisms that are optimized for the processing and encoding of the particular cue. Subsequent evolutionary changes in physical configurations (i.e., changes in head size and/or hearing range) might force the use of additional cues. However, the neuronal mechanisms and coding principles that will be employed to process the additional binaural cue is determined by the original mechanisms/principles that are already in place. Thus, while the same binaural cues (e.g., ITD) are used for sound localization by birds and mammals, their evolutionary histories – and hence neuronal mechanisms – are of different origin.

### A SCENARIO FOR THE EVOLUTION OF SOUND LOCALIZATION IN BIRDS: ITD AS THE ORIGINAL BINAURAL CUE

**FIGURE 3:**
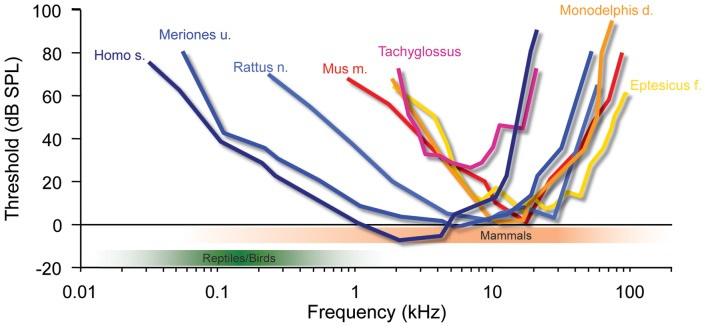
**Mammalian hearing originated in the ILD-dominated range.** A hallmark of mammalian audiograms is that they are centered in the high-frequency range (>10 kHz), where ILDs are the dominant cue for sound localization. Many recent mammalian species like mice (*Mus m*.), bats (*Eptesicus f*.), rats (*Rattus n*.) and short-tailed opossums (*Monodelphis d*.) even expanded the high-frequency hearing compared to early mammals (*Tachyglossus*), allowing for an increase in obtainable ILDs. Only few species including Gerbils (*Meriones u.*) and man (*Homo s*.) expanded their hearing range into the low-frequency range, where ITD is an attainable sound localization cue (<2 kHz). Audiograms modified from: Echidna/Tachyglossus: Mills and Shepherd (2001); *Monodelphis*: Reimer (1995); Mouse: Heffner and Masterton (1980), Radziwon et al. (2009); Bat (*Eptesicus fuscus*): Koay et al. (1997); Rat (hooded rat): Heffner et al. (1994); Gerbil: Ryan (1976).

As pointed out above, we cannot yet say with certainty how often tympanic ears evolved in sauropsids. Currently, there is conflicting evidence between morphological and molecular studies on whether testudines represents a sister group of archosaurs or whether they are more remote and thus developed tympanic ears independently (Hedges, 2012). Moreover, if tympanic ears evolved only in the Triassic (Clack, 1997) and archosaurs first appeared in the Late Permian or Early Triassic (Gower and Sennikov, 1996), their organs for detecting air-borne sounds may even have been acquired separately from other diapsids in that group. In any case, almost all early archosaurs were quite large (compared to early mammals, see below) and increased in size during the Triassic (e.g., crocodiles and dinosaurs) to give rise to the largest land-dwelling tetrapods (Gower and Weber, 1998). Birds inherited their tympanic ears from them (e.g., dinosaurs). Like all archosaurs they possessed only one middle-ear bone, the columella (derived from the hyomandibular bone, like the stapes in mammals), a fact which, by and large, limits their audiograms to relatively low frequencies (from a few 10s of Hz to a few kHz; green shaded area in **Figure 3**; Fleischer, 1978; Rosowski and Saunders, 1980). Additionally, a connection between the two middle-ear cavities via a thin tube makes their hearing system a kind of pressure-gradient receiver (reviewed in Manley, 2010) that creates interferences and thereby moderately enhances ITDs. For instance, in young chicks maximal ITDs can be enhanced to reach up to 180us for low-frequency sounds, whereas maximal ITDs reach only 100 μs at frequencies of 2–4 kHz and thus appear to rely solely on the interaural distance (Hyson et al., 1994). Hence, Triassic archosaurs perceived low frequencies associated with only minimal ILDs, but experienced comparatively large ITDs (up to several 100 μs, in some dinosaurs well above 1 ms). It is therefore not surprising that these animals developed a sophisticated neuronal ITD coding system [e.g., the nucleus laminaris, NL; (Carr, 1993; Carr et al., 2001)]. Interestingly, testudines (turtles) possess a prominent NL (Willis et al., 2013), corroborating the molecular evidence that they might be closely related to archosaurs (Shen et al., 2011; Chiari et al., 2012; Lu et al., 2013; Field et al., 2014). The situation is less clear for other diapsids, apart from the fact that they have a true pressure-gradient receiving system (the middle-ear cavities are continuous with the oral cavity), which introduces significant binaural interference patterns that will generate a mixture of ITDs and ILDs of their own (Christensen-Dalsgaard and Manley, 2008; Christensen-Dalsgaard, 2011; Christensen-Dalsgaard et al., 2011). To summarize, the sound localization system in birds most likely evolved to process low-frequency signals and thus is specialized for ITD detection.

### A SCENARIO FOR THE EVOLUTION OF SOUND LOCALIZATION IN MAMMALS: ILD AS THE ORIGINAL BINAURAL CUE

The ancestors of mammals, which belonged to the late therapsids, were probably the last to develop tympanic ears in the Late Triassic (Allin, 1975; Clack, 1997). Two factors distinguish their evolution from all others. Firstly, they developed a secondary jaw joint, probably due to a change of diet to seeds, which required crushing (Crompton, 1963; Kemp, 1982). This deprived all three original jaw ossicles of their former primary function – cutting and tearing – and allowed them to take on a secondary function, the transmission of sounds (substrate sound/bone conductance from the lower jaw). Secondly, at this point, therapsids – the hitherto dominant group of tetrapods – were being pushed aside by the rapidly evolving dinosaurs, and were facing extinction. The only clade that survived did so by rapidly decreasing in size, ultimately giving rise to animals smaller than laboratory mice. Interestingly, during this phase, the originally much larger middle-ear bones shrank isometrically with the rest of the skull to a size suitable for transmitting sounds [for instance *Thrinaxodon* (Estes, 1961)], – and they have allometrically remained in this state despite the ensuing changes in overall body size (Crompton and Hylander, 1986; Maier, 1990). Early mammals like *Morganucodon* (early Jurassic) give us insight into their nocturnal life in the shadow of the dinosaurs. They possessed the new three-ossicle middle-ear, which was basically identical to that of today’s echidnas (*Tachyglossus*, one of the two recent monotremes). Theoretical considerations had already suggested that this ear does not efficiently transmit low frequencies, but responds well to mid-range frequencies from a few up to maximally 20 kHz (**Figure 3**; Rosowski and Graybeal, 1991). More recent psychoacoustic evaluations and auditory brainstem potential measurements in echidna support this assumption (Mills and Shepherd, 2001). Hence, early mammals like *Morganucodon* lived in a different acoustic world from that inhabited by the dominating diurnal reptiles. This is also suggested by their small size and that of a potential larynx, which would have produced high-frequency sounds and is supported by the fact that most small mammals still use communications calls in a frequency range beyond that of reptilian and bird hearing (e.g., mother – pup communication, Liu et al., 2003; Ehret, 2005). This separation between reptilian (and later, bird) hearing and that of mammals has apparently tended to increase rather than diminish during evolution. Small marsupials (like *Monodelphis*) or placental mammals (mice, bats etc.) extended their hearing range, as evident from fossils showing the coiled cochlea as a result of lengthening (Fernández and Schmidt, 1963). Comparing the audiograms of recent mammals of various groups indicates that their hearing range almost exclusively extended into the high-frequency range (**Figure 3**). Bat echolocation calls mostly fall within the hearing range of small mammals and should not be considered as unusual – “ultrasound” is a purely anthropocentric, not a mammaliocentric term (**Figure 3**, although this does not imply that bats are not highly specialized in other ways, and some species further extended their hearing range even above 100 kHz). Notably, such extension of hearing range to ever higher frequencies significantly improves the use of HRTFs in the vertical plane. Since localization in the vertical is of the utmost importance for small prey animals (Wallace et al., 2013), reliable HRTF-based localization may well have been a crucial evolutionary pressure on the hearing range of small early mammals. The second advantage of mainly high-frequency hearing is that even the smallest mammals have always experienced significant ILDs (**Figure 2**; Erulkar, 1972; Harnischfeger et al., 1985). On the other hand, their tiny heads produced ITDs of maximally a few 10s of μs (**Figure 2**, <50 μs in animals like *Morganucodon*). There is ongoing debate about whether early mammalian ears also acted as pressure receivers, which could have increased the range of ITDs by a few 10s of μs (Köppl, 2009; Manley, 2010). Whether this would have been significant enough to justify the use of ITDs (despite the availability of large ILDs) seems doubtful. And even if it were, one may ask why such a useful feature would have disappeared in all mammals (including monotremes)? In both cases, the conclusion appears obvious: mammals simply did not need to process ITDs.

Even today, most small mammals rely almost entirely on ILDs, and the neuronal structure responsible for the initial processing of ILDs, the lateral superior olive (LSO), is homogenous in all terrestrial mammals investigated (Tollin, 2003; Grothe et al., 2010). In contrast, the ITD processing structure, the medial superior olive (MSO) exhibits significant differences in shape and size, which are likely to be related to the hearing range in the respective species (low- versus high-frequency sensitivity; Grothe, 2000). Significant selection pressure to use ITDs existed only relatively late during the evolution mammals, probably in relation to increasing body size, which not only conditioned production of low-frequency communication calls, but also necessitated larger territories and long-distance communications – and low frequencies travel further.

## THE FUNCTION OF INHIBITION IN ILD PROCESSING CAN EXPLAIN ITS ROLE IN ITD PROCESSING

### ILDs AS A STARTING POINT FOR A POPULATION CODE OF SPATIAL POSITION

**FIGURE 4:**
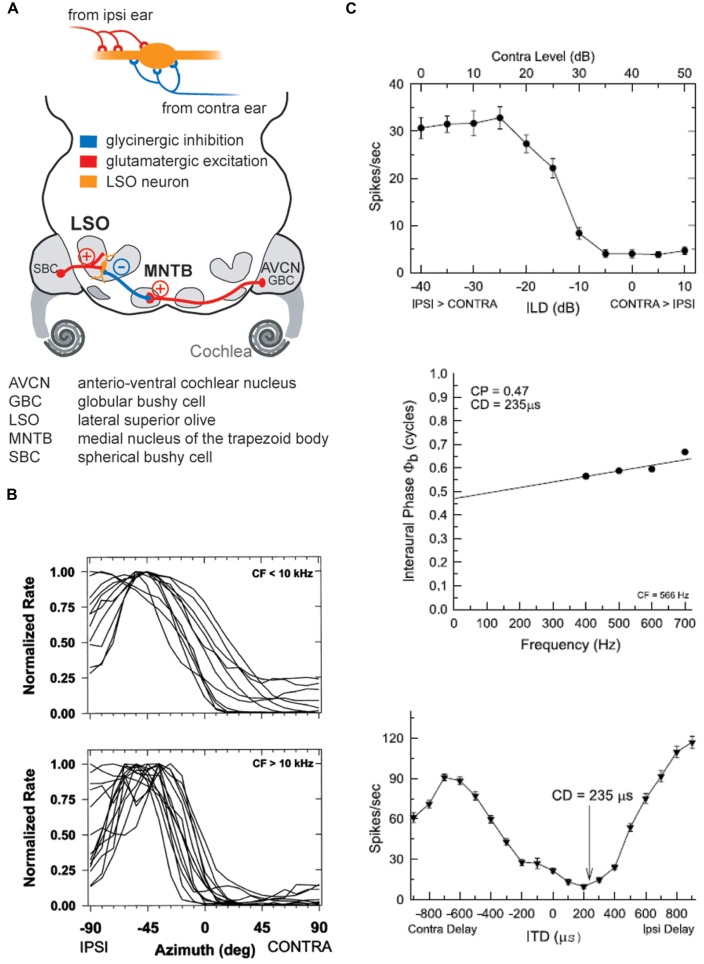
**The coincidence mechanism of LSO neurons allows both as ILD and ITD detection. (A)** LSO neurons receive excitatory inputs from SBCs in the ipsilateral AVCN and inhibitory inputs from the MNTB that is innervated from by GBCs from the contralateral AVCN. **(B)** The spatial tuning functions of LSO neurons take a hemispheric shape with the slope of the functions crossing frontal azimuthal positions. Upper and lower panels show normalized tuning functions for LSO neurons in cat with CFs below and above 10 kHz, respectively, recorded under virtual acoustic space stimulation that incorporates the HRTFs. Re-printed with permission from Tollin and Yin (2002). **(C)** Low CF neurons in the LSO are both ILD and ITD sensitive: upper panel shows ILD tuning function of a cat LSO neuron (CF = 566Hz), while the lower two panels illustrate the ITD-sensitivity of the same neuron. Note that the characteristic delay (CD) for this neuron, i.e., the delay of coincidence of excitatory and inhibitory inputs, results in a minimal response rate. Re-printed with permission from Tollin and Yin (2005).

As outlined above, early mammals most probably could hear high-frequency sounds and had relatively small heads. Hence, ILDs were the only binaural cues available to them for azimuthal sound localization. This suggests that the ancestral neuronal structure used to process binaural spatial information was devoted to ILD detection. It is well established that ILD sensitivity is generated by the LSO in the brainstem, whose bipolar neurons are the initial site of binaural convergence (Galambos et al., 1959; Boudreau and Tsuchitani, 1968; Tsuchitani and Boudreau, 1969). They integrate excitatory (glutamatergic) inputs from the ipsilateral antero-ventral cochlear nucleus (AVCN) with inhibitory (glycinergic) inputs coming from the ipsilateral medial nucleus of the trapezoid body (MNTB), which itself is innervated by the contralateral AVCN (**Figure 4**). This integration process can be thought of as a comparative mechanism that gages the relative sound levels at the two ears (within a particular spectral bandwidth at a given time point), which are encoded in the respective activity levels of the two LSO inputs (Moore and Caspary, 1983; Finlayson and Caspary, 1989; Sanes, 1990; Tollin, 2003). Accordingly, LSO response rates (measured as the number of action potentials elicited per unit time) are highest for ipsilateral sound-source locations that create positive ILDs, i.e., high sound level at the ipsilateral ear allows the excitatory pathway to be fully activated, whereas the sound level at the farther ear is greatly attenuated by the skull, and thus activation of the contralateral inhibitory pathway is minimal. More importantly, response rates are faithfully modulated as a function of the ILD, and most LSO neurons are completely inhibited from spiking at ILDs favoring the contralateral ear (negative ILDs). Such ILD response functions typically take the shape of a sigmoid, generating high sensitivity for small changes in ILD along the slope of the function (**Figure 4**). Note that any ILD sensitivity found in downstream brain areas crucially depends on an LSO input, be it excitatory or inhibitory. This is most probably attributable to neuronal specialization necessary for ILD extraction (see below).

The LSO has no homolog in other vertebrates. In birds, ILD sensitivity is generated by convergence of contralateral excitatory and ipsilateral inhibitory inputs at the level of the lateral lemniscus (Moiseff and Konishi, 1983; Takahashi and Keller, 1992). This connectivity therefore represents a rather complex reciprocal ILD processing circuit that does not reflect the integration mechanism of monaural inhibition and excitation of the mammalian LSO: first, inhibitory and excitatory ear are reversed. Second, the ipsilateral inhibition is conveyed by the lateral lemniscus of the other hemisphere, hence by a binaural nucleus. Third, because it is conveyed via an additional synaptic station through the binaural detector of the other hemisphere, inhibition is significantly delayed relative to excitation and seems to serve a response gain modulation (Steinberg et al., 2013). As we will explain in the following and in 3.2., inhibition in the LSO has purposes directly related to establishing binaural sensitivity.

The LSO is well-developed in all terrestrial mammals, including echo-locating bats and humans (Moore, 2000). The overall size of the LSO in a particular species seems to correlate with the range of frequencies to which that species is sensitive (Moore, 2000), most probably owing to the tonotopic organization of the nucleus. All mammals appear to use the same neural mechanism for processing ILDs in the LSO, namely the integration of ipsilateral excitatory inputs from the AVCN and contralateral, inhibitory inputs via the MNTB (see below, Grothe, 2000; Yin, 2002; reviewed in Grothe et al., 2010). Hence, they employ similar coding strategies for high-frequency sound-source localization at the level of the LSO. This coding strategy can be described as a roughly hemispheric code in which individual neurons encode a range of ILDs through response-rate modulation along the slope of their ILD functions (Tollin and Yin, 2002). The LSO neurons studied to date exhibit rather similar ranges of sensitivity to ILDs: the slope of LSO ILD functions is typically centered close to 0 dB ILD (Park et al., 1997, 2004; Park, 1998; Tollin and Yin, 2002). Interestingly, in a study of the cat LSO using virtual space stimuli (i.e., incorporating monaural spectral effects of sound-source location), Tollin and Yin (2002) observed that the tuning of LSO neurons is remarkably stereotypic, as the slope of most spatial-response functions covered a similar range of azimuthal space around the midline and the nearby ipsilateral areas (**Figure 4**). Together, these findings suggest an overrepresentation of near-midline locations, in agreement with the reported maximal psychophysical resolution of ILDs around the midline (Blauert, 1997). However, the interpretation of characteristics of ILD functions in general is difficult, as the peak and slope positions of ILD functions are markedly affected (shifted) by previous activity levels (Park et al., 2008). These shifts are mediated by, among other mechanisms, the retrograde release of GABA from LSO cells onto their own presynaptic inputs (Magnusson et al., 2008), which suggests high plasticity of ILD coding based on recent stimulus history. Hence, even at the level of single binaural comparator neurons, representations of spatial positions are likely to change according to the current auditory context rather than being inflexibly coded. This use of inhibition to generate flexible representations, and their implications for downstream coding, are discussed in more detail in Section “Dynamics of ILD and ITD Processing: GABA_B_-Mediated Inhibition” below.

### ILD PROCESSING – THE ROLE OF THE MNTB AND GLYCINERGIC INHIBITION

The integration of inhibitory and excitatory inputs by LSO cells is often informally referred to as subtraction. This overly simplistic analogy should be treated with caution, insofar as it tends to imply the comparison of net activity levels in the ipsi- and contralateral input integrated over the entire duration of a given acoustic stimulus. In fact, essentially the opposite is true, as timing information – more specifically, information relating to temporal fluctuations in stimulus amplitude – is highly conserved within the LSO circuit. Indeed, neurons involved in ILD detection, including the components of the inhibitory MNTB pathway, are among the most temporally precise in the brain. Two key demands on the system impose the need for high temporal acuity.

The first is the general functional requirement for high temporal resolution in sound localization circuits. These systems cannot afford to integrate over long intervals to produce an average intensity difference, because the source of this average signal might well have changed in the meantime. Moreover, in the presence of multiple, concurrently active sound-sources, short integration times are crucial for discrimination between individual sounds (Meffin and Grothe, 2009; Khouri et al., 2011). Natural signals (communication calls, speech, rustling noises generated by moving prey, etc.) are characterized by prominent and rapid amplitude modulations. Hence, to faithfully detect and track the site of origin of such signals, the LSO circuit must be able to resolve ILDs on very short temporal scales (Joris and Yin, 1995; Tollin, 2003). This is accomplished by the well-known phenomenon of phase-locking, which describes the ability of auditory (brainstem) neurons to lock the timing of their spiking activity to a particular phase of the stimulus (Joris et al., 1994). Phase-locking is commonly invoked in the context of low-frequency carrier or envelope sinusoidal signals, but can (and should) be generalized to the encoding of the rising slopes or transients in any complex signal, irrespective of its frequency (Dietz et al., 2014). Accordingly, phase-locking allows for the precise encoding of a particular time of occurrence in the auditory nerve and downstream pathways. The classical work of Joris et al. (1994) has demonstrated that the quality of phase-locking (measured in terms of vector strength) is actually maintained or even enhanced in the post-synaptic target of the auditory nerve fibers of the binaural system, namely the spherical and globular bushy cells (SBCs and GBCs) of the AVCN, which provide the input to MNTB and LSO, respectively (Warr, 1966; Spangler et al., 1985; Cant and Casseday, 1986; Friauf and Ostwald, 1988; Sanes, 1990). In particular, synaptic transmission between GBC axon and MNTB soma has been studied extensively because of the large size of the pre-synaptic structure (Schneggenburger and Neher, 2005; Kopp-Scheinpflug et al., 2011; Borst and Soria van Hoeve, 2012), and this synaptic relay is one of the fastest and temporally most precise known in the brain (von Gersdorff and Borst, 2002). Evidently, MNTB neurons exhibit similar phase-locking precision to GBCs, while LSO cells – the post-synaptic targets of both MNTB and SBCs – themselves exhibit fast membrane kinetics that allow for exquisite temporal sensitivity to the arrival time and duration of incoming synaptic events (Tollin, 2003). Taken together, these properties of the LSO circuit allow for highly precise and independent ILD processing of each fast transient or onset in a signal.

The second functional demand that necessitates the extreme temporal sensitivity of the LSO circuit is directly linked to the previous argument and explains the specific morphological and physiological adaptations for temporal fidelity and transmission speed that are found within the inhibitory sub-circuit involving the MNTB. A faithful representation of amplitude-modulated signals requires not only that both the excitatory and inhibitory inputs should reliably encode the precise time of occurrence of transient events, but also that both inputs should arrive in close coincidence at the LSO cell to allow for interaction of the two. Clearly, this poses a challenge for the inhibitory input, as it must somehow compensate for the longer axonal pathway from the contralateral AVCN, as well as for the additional synapse between GBC and MNTB, which will introduce a further delay. Indeed, a detailed anatomical examination of the MNTB pathway reveals particular specializations for high conduction velocity, as both axon diameter and myelin thickness are larger in GBCs than in SBCs (Morest, 1968; Schwartz, 1992). Moreover, synaptic delays at the calyx of Held are among to the shortest that have been measured in the CNS (von Gersdorff and Borst, 2002; Kopp-Scheinpflug et al., 2011). Accordingly, physiological evidence shows that inhibition is capable of suppressing even the first spike of LSO responses (Tsuchitani, 1988; Tollin, 2003), demonstrating (at least) coincident arrival of contralateral inhibition and ipsilateral excitation.

In summary, the ILD circuit represents the ancestral binaural sound localization circuit of mammals. LSO neurons detect ILDs via a coincidence detector mechanism of ipsilateral excitation and contralateral inhibition. All components of the LSO circuit are tuned for temporal fidelity, and the inhibitory pathway of the MNTB in particular has evolved anatomical and physiological adaptations to compensate for the longer pathway and additional synaptic delay.

### ITD PROCESSING IS DERIVED FROM ILD PROCESSING IN MAMMALS

#### Shared components of ILD and ITD circuits

**FIGURE 5:**
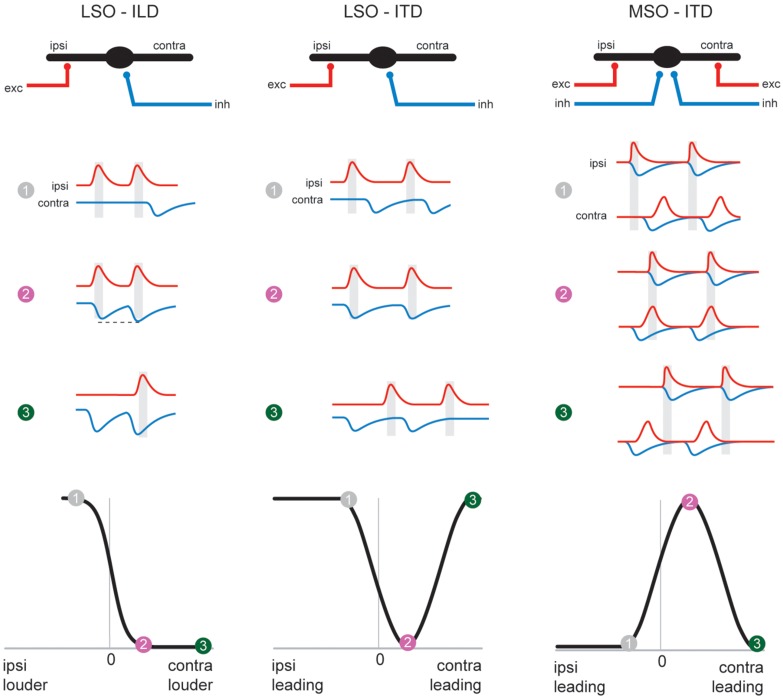
**The MSO coincidence mechanism is derived from the LSO coincidence mechanism.** The schematic depicts temporal relationships of EPSPs and IPSPs, (red and blue traces, respectively) during ipsi-favoring (1, gray), slightly contra-favoring (2, magenta) and strongly contra-favoring (3, green) input combinations. The left-hand and middle column illustrates processing of these synaptic inputs in the LSO for ILDs and ITDs respectively, and ITD processing in the MSO is shown in the right-hand panel. Note that the MSO integrates EPSPs and IPSPs from both the ipsi- and contralateral side, because of the additional excitatory (contralateral) and inhibitory (ipsilateral) inputs compared to the LSO. The panel in the lower row explains how conditions 1–3 affect spatial tuning functions in the respective nuclei.

**FIGURE 6:**
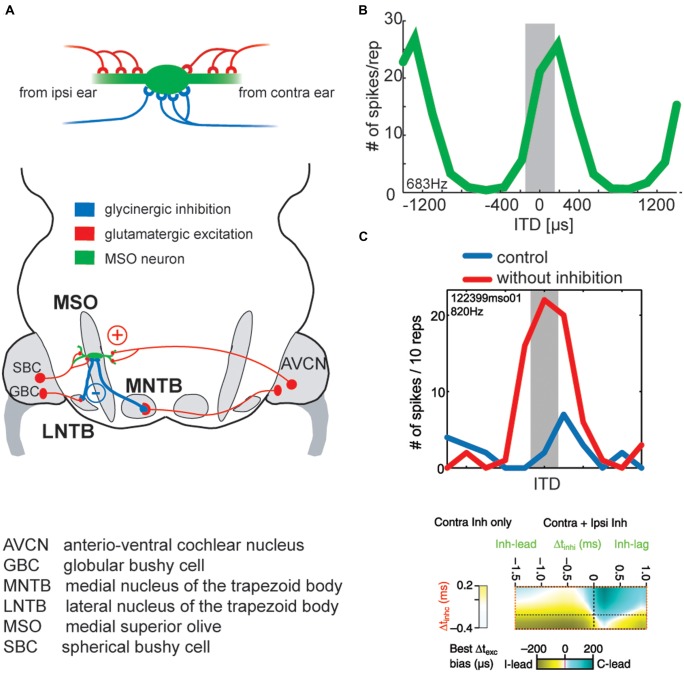
**Binaural excitation and inhibition of the MSO circuit allows fine-tuning of the coincidence mechanism. (A)** MSO neurons receive binaural excitatory inputs from SBCs in the AVCN of either side and binaural inhibitory inputs from LNTB and MNTB, which are innervated by GBCs of the ipsilateral and contralateral AVCN, respectively. **(B)** ITD tuning function of a gerbil MSO neuron (CF = 683Hz). Note that the peak of the function (“best ITD”) is positioned at a contralateral leading ITD outside of the range of physiological ITDs (gray area), while the slope spans the entire range of physiological ITDs. **(C) Upper panel**: blocking inhibition in MSO cells *in vivo* shifts the best ITD toward 0 ITD. Thus, inhibitory inputs tune the ITD of coincidence in MSO cells. Taken from Pecka et al. (2008). **Lower panel**: combination of ipsi- and contralateral inhibitory inputs (right-sided box) allow for both larger shifts of the best ITD (color-coded) than contralateral inhibition alone (left-sided box). Modified from Myoga et al. (2014).

The evolutionary and anatomical evidence suggests that, as a nucleus for highly precise binaural discrepancy detection in the time domain, the MSO might have evolved in response to other morphological adaptations that occurred within Mammalia (see The Origins of Spatial Hearing). Increased body (and head) size resulted in a larger interaural distance and a larger larynx, and made it possible to communicate over larger distances (which are best bridged by low-frequency signals). These constraints in turn exerted a selective pressure which favored adaptations that allowed for processing of ITDs (Grothe, 2000; Schnupp and Carr, 2009), as more informative and reliable cues with which to localize relevant sounds or communication calls (since ILDs are negligible at low frequencies). Coincidentally, the “subtraction” mechanism embodied in the LSO, which had developed for ILD detection, is already equipped (pre-adapted) for ITD detection. As has been demonstrated by studies in both cats and chinchillas (**Figure 4**; Finlayson and Caspary, 1991; Joris and Yin, 1995; Tollin and Yin, 2002, see also Park et al., 1996), response rates of low-frequency LSO neurons are strongly modulated by microsecond changes in ITD. These data therefore clearly establish that the temporal fidelity of the glycinergic MNTB input is sufficient to generate ITD sensitivity in LSO neurons tuned to low frequencies by modulating the excitatory inputs excitatory post-synaptic potentials (EPSPs) in response to fast transients. Hence, the MSO circuit which, in mammals specialized for hearing low-frequency sounds, is dedicated to ITD processing only, can be conceptually regarded as a refined LSO circuit (**Figure 5**). Interestingly, mammals with good low-frequency hearing typically possess both a large low-frequency limb of the LSO and a well-developed MSO (Grothe, 2000; Grothe et al., 2010). Potentially their combined output is beneficial to the reliable encoding of sound-source positions, because the spatial tuning functions in the two nuclei are mirror images of each other: a purely suppressive coincidence mechanism (i.e., spiking occurs *unless* binaural coincidence exists) in the LSO is converted into an essentially excitatory coincidence mechanism for the MSO (spiking occurs *only if* binaural coincidence exists). This conversion is achieved by the addition of two more inputs onto MSO neurons. First, a second excitatory input from the contralateral side is required to allow for binaural excitatory coincidence detection. Second there is also an additional inhibitory ipsilateral input via the LNTB (**Figures 5 and 6**). Thus, synaptic inhibition represents an essential feature of the MSO circuit. Anatomical and physiological studies have demonstrated that MSO neurons receive relatively few, but unusually strong, glycinergic inputs (Clark, 1969; Grothe and Sanes, 1993, 1994; Kapfer et al., 2002; Werthat et al., 2008; Couchman et al., 2012) that are well balanced in quantity and quantal size with the excitatory MSO inputs (Couchman et al., 2010). Consequently, the MSO must integrate excitatory and inhibitory inputs from both sides, and is not a simple excitatory coincidence detection circuit. The evolutionary pressure that favored such an arrangement is unclear, but it is reasonable to speculate that the system requires a delicate balance of excitation and inhibition in order to accomplish precise temporal integration (see below). Functionally speaking, the two inhibitory inputs may have important implications for the specific ITD tuning of MSO neurons, a topic that is still being debated today. Initially, research focused on the contralateral source of inhibition via the MNTB, as it had been much more extensively characterized (see above). We suggested earlier that the basic role of phase-locked inhibition might actually be the fine-tuning of best delays (ITD of maximal spiking response) in MSO neurons by modulating the time window for binaural excitatory inputs (**Figure 6**; Brand et al., 2002; Pecka et al., 2008). Specifically, it was suggested that contralateral inhibitory post-synaptic potentials (IPSPs) might arrive at the MSO cell soma slightly in advance of the contralateral EPSPs (**Figure 5**). This would result in a delay of the net excitatory potential, and thus explain the clustering of contralateral best delays in MSO neurons, which has been observed experimentally and across species (McAlpine et al., 2001; Hancock and Delgutte, 2004; Pecka et al., 2008). This scenario might seem to impose significant temporal demands on the MNTB input, but – as described earlier – it is well established that the same MNTB input suppresses the onset of LSO responses, i.e., that contralateral inhibition arrives simultaneously with the ipsilateral excitation. Thus, it seems plausible that contralateral inhibition should actually be slightly faster than contralateral excitation, and both older and recent work with acute brain slice preparations has confirmed that IPSPs can precede EPSPs at MSO cell somata after contralateral AVCN stimulation (Grothe and Sanes, 1994; Roberts et al., 2013). However, the influence of contralateral inhibition alone is insufficient to explain the extent of modulation suggested by *in vivo* pharmacological experiments (Zhou et al., 2005; Jercog et al., 2010; Roberts et al., 2013; van der Heijden et al., 2013). To clarify this issue, we recently investigated the ability of the ipsilateral source of inhibition to modulate coincidence detection in the MSO, and found that it had a marked capacity to modulate the timing of binaural coincidence (**Figure 6**; Myoga et al., 2014). Although more research is required to thoroughly understand the ipsilateral source of inhibition (Leibold, 2010), it is becoming increasingly clear that having two sources of inhibition (instead of just the contralateral source) provides for greater flexibility in modulating the circuit (**Figure 6**). Thus, while inhibition in the MSO circuit remains a topic of debate, it is reasonable to assume that it serves a central function in the circuit: compared to the ITD-sensitive archetype (i.e., the LSO), an additional (ipsilateral) inhibitory input has evolved in the MSO circuit.

Dedicated inhibitory pathways also exist within the NL-circuit in chicks and owls that seems to serve multiple functions related to ITD processing (Burger et al., 2011). Neurons of the superior olivary nucleus (SON) provide GABAergic inputs to the NL and form a gain control circuit by reducing the amplitude of excitatory inputs and shortening their duration, thereby ensuring consistent ITD sensitivity across intensity levels (Peña et al., 1996; Dasika et al., 2005; Nishino et al., 2008). SON-mediated inhibition also improves phase-locking precision of both the excitatory inputs and NL responses (Nishino et al., 2008; Burger et al., 2011). Importantly, the inhibition from SON onto NL neurons is not timed (it is decoupled from the phase-locked excitation; Yang et al., 1999), and it actually has a depolarizing effect on the NL cells (due to a high intracellular Cl^-^ concentration), which in turn activates low-threshold potassium-channels that lead to shunting of the cell (Hyson et al., 1995; Yang et al., 1999; Burger et al., 2005a). Interestingly, phase-locked GABAergic inhibition that is conveyed by a feed-forward circuit outside the SON has been found to act on NL neurons tuned to very low frequencies to cooperatively enhance ITD tuning together with tonic inhibition (Yamada et al., 2013). It follows that, analogs to the mammalian system, ITD processing at low frequencies requires the (co)-action of timed inhibition. Hence, both in the mammalian and avian ITD system, inhibition serves a prominent function toward refining the ITD sensitivity of the detector neurons. However, the respective neurotransmitters and their associated mode of action and functional time scales are different.

#### Shared components in ILD and ITD circuits lead to shared coding principles

**FIGURE 7:**
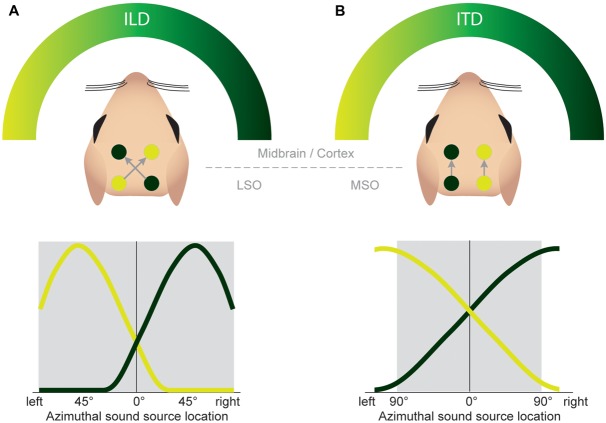
**Both the ILD and ITD code is based on hemispheric tuning functions.** The azimuthal tuning function of both LSO and MSO span a wide range of azimuthal space. **(A)** LSO neurons respond best to ipsilateral sound-source positions (compare **Figure 4B**). This ipsi-preference is flipped to a contra-preference upstream of the LSO because of the contralateral projections of LSO neurons to the midbrain. **(B)** MSO neurons respond best to contralateral sound-source positions. This contra-preference is maintained upstream of the LSO because of the ipsilateral projections of MSO neurons to the midbrain.

So far, we have discussed the similar roles of MSO and LSO as binaural discrepancy detectors that share many of their circuit components and design principles. Consequently, similarities are also found in the ways in which particular ITDs and ILDs are reflected in the spiking responses of the respective neurons. Both MSO and LSO neurons exhibit broad, hemispheric tuning to sound-source location, i.e., response rates change monotonically over a large range of azimuthal space (Grothe et al., 2010). Importantly, spatial tuning in both nuclei appears to be more or less stereotypical, with the majority of neurons having their highest spatial sensitivity (the slope of their tuning functions, which conveys most information about changes in location) at frontal positions (**Figures 4 and 6**; Tollin, 2003; Harper and McAlpine, 2004). For ILDs, this stereotypical arrangement becomes most apparent when (virtual) free-field stimulation is used, suggesting a crucial role for spectral composition of the stimuli in azimuthal sound localization at higher frequencies (Tollin and Yin, 2002). Furthermore, the peak positions of ITD tuning functions have been found to depend on stimulation frequency in many species, irrespective of the head sizes of the species studied (Middlebrooks et al., 1994; McAlpine et al., 2001; Hancock and Delgutte, 2004; Pecka et al., 2008; Werner-Reiss and Groh, 2008). These data have multiple crucial implications: first, that hearing range and the presence of a well-developed MSO, and not body or head size (i.e., physiological ITD range), determines whether a particular species exploits ITDs for sound localization. Since the MSO circuitry is similar in all mammals with low-frequency hearing (Grothe, 2000), neuronal microsecond ITD sensitivity (not spatial acuity in degree, which is a function of interaural size) is also similar across species (Phillips et al., 2012). Second, the frequency-dependence of ITD tuning refutes the notion of a distributed labeled-line representation of azimuthal space (i.e., in which the activity of individual neurons represents the reception of a signal from a fixed direction in space), and have led to numerous speculations on the nature of the underlying coding strategy. The broad tuning functions stimulated the idea of hemispheric, oppositely coding channels on each side of the brain that might be compared upstream of the MSO and LSO (McAlpine et al., 2001; Stecker and Middlebrooks, 2003; Hancock and Delgutte, 2004; Harper and McAlpine, 2004; **Figure 7**). Note that the MSO output – but not the LSO output – to the midbrain crosses the midline, which unifies the hemispheric polarity of the two within each midbrain side (**Figure 7**). While the particular nature of this code is currently under debate (Day and Delgutte, 2013; Goodman et al., 2013), one compelling concept relies on the idea that similar activity levels in each channel represent sound-source position at the midline, such that a relative increase in activity in one of the two brain hemispheres would indicate a proportionally contralateral location with respect to the more active brain hemisphere (**Figure 7**). Psychophysical and functional imaging studies corroborate this scenario of hemispheric coding also in humans (Thompson et al., 2006; von Kriegstein et al., 2008; Magezi and Krumbholz, 2010; Salminen et al., 2010). In particular, using elegant adaptation paradigms, Phillips and Hall (2005), Vigneault-MacLean et al. (2007) have confirmed the presence of a population code that underlies sound localization in humans and also showed that prior stimulation influences subsequent spatial perception. These data pointed the way to more recent discoveries pertaining to how spatial tuning functions can be strongly modulated according to their recent acoustic context. Physiologically, such activity-dependent effects have been demonstrated in the cortex and midbrain (Dahmen et al., 2010; Lee and Middlebrooks, 2011), and even in the MSO and LSO (Magnusson et al., 2008; Park et al., 2008; Stange et al., 2013), suggesting that the primary role of MSO and LSO might not be the encoding of absolute sound-source positions in space (in contrast to the avian system, which employs a labeled line code with a consequently sparse output corresponding to any one position in space), but rather of their relative locations compared to other sound-sources. Similar adaptive coding concepts are well-known in other sensory modalities and will be considered in the context of sound localization in the following section.

## DYNAMICS OF ILD AND ITD PROCESSING: GABA_B_-MEDIATED INHIBITION

**FIGURE 8:**
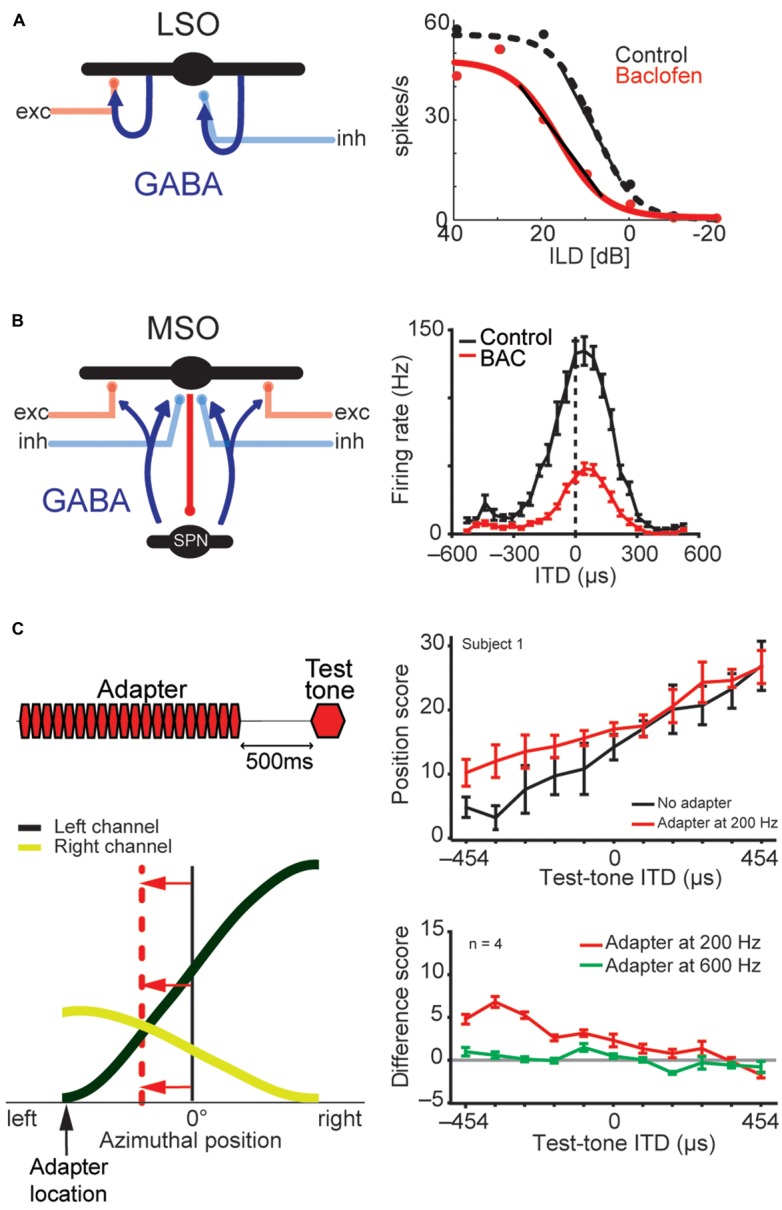
**LSO and MSO responses are modulated by prior activity and thus encode relative sound-source positions.** The firing rates of both LSO and MSO neurons are controlled in an activity-dependent manner via GABAergic inhibition. **(A)** In the LSO, GABA is released retrogradly by dendritic release from LSO neurons onto their synaptic partners. **(B)** In the MSO, GABA is released via a di-synaptic feedback loop including the SPN (superior para-olivary nucleus). Both mechanisms generate levels of GABA-mediated inhibition that are proportional to the prior activity of the respective LSO/MSO neuron. In both cases, the firing activity is modulated by GABA_B_-receptors, resulting in a divisive gain control mechanism. Data modified from Magnusson et al. (2008) **(A)** and Stange et al. (2013) **(B)**. **(C)** *In vivo* recordings in gerbils showed that upon prolonged presentation of an Adapter stimulus from a very lateralized sound-source position (stimulus paradigm is schematized in upper left), the GABAergic gain control results in asymmetric changes in the two coding channels (ipsi- and contralateral MSOs or LSOs) due to the asymmetric activity profile between the two channels and the activity dependence of the gain control mechanism. Particularly, the cross-point between left and right coding channel is shifted away from the actual midline (indicated by red horizontal arrows) and toward the location of the Adapter location (indicated by black vertical arrow). In accordance with the hypothesis of hemispheric coding channels of sound localization, this stimulation paradigm leads to systematic shifts of the perception of test tone positions in human listeners (right column). The result from a single subject is shown in the upper panel, the average from four subjects is shown in the lower panel. A difference score of 5 approximates a shift in lateral perception of 30°. As predicted from the activity-dependence of the GABAergic gain control circuit, the presentation of an Adapter stimulus at a different frequency than the test tone (green line) did not affect the localization percept. Data taken from Stange et al. (2013)

Across sensory modalities, the representation of stimulus features in an individual coding element may depend on the global distribution of that feature across the population of coding channels. For example, for a given magnitude of a feature, the response rate of individual neurons is modulated by the overall distribution of response rates in the entire population of neurons. This adaptation principle, in which the tuning of single neurons is normalized to the population average, serves to prevent response saturation and thereby increases coding efficiency (Wark et al., 2007; Carandini and Heeger, 2012). Traditionally, such adaptive strategies have not been associated with sound localization. However, recent results from both human psychophysics (Getzmann, 2004; Phillips and Hall, 2005; Vigneault-MacLean et al., 2007; Maier et al., 2009; Dahmen et al., 2010) and animal physiology (Park et al., 2008; Dahmen et al., 2010) have clearly demonstrated a dependency of ITD and ILD tuning, and even spatial perception, on the prior stimulation (specifically, on the stimulus properties and their temporal profile). We have recently discovered similar dynamic adaptation mechanisms in both the LSO and MSO directly (Magnusson et al., 2008; Stange et al., 2013). In both nuclei, the response magnitude of individual neurons is causally correlated with its prior spiking activity. High prior activity leads to strong response adaptation (decreased rates) and *vice versa*. Notably this particular response modulation, which acts on time scales of 10s of ms and can last for seconds, is mediated by similar mechanisms but employs different circuits in MSO and LSO. In each case, GABA_B_ receptor signaling is involved, albeit in distinct ways (**Figure 8**). In the LSO, GABA is released in activity-dependent manner directly from principal LSO neurons and differentially activates GABA_B_ receptors that are located on its presynaptic inputs (Magnusson et al., 2008, the effect is stronger on excitatory than on inhibitory inputs). Thus, the strength of inputs to a given LSO cell is controlled by its own spiking activity. In contrast, MSO cells are not themselves GABAergic. Instead, a di-synaptic feedback-loop exists, in which MSO neurons innervate GABAergic neurons in a nearby nucleus that subsequently feed back onto the MSO (Stange et al., 2013; **Figure 8**). While it is not known how specific the projections, and thus the adaptation effects, are between the two nuclei, one-to-one cell connectivity seems improbable (GABA is most probably released via volume transmission). Hence this circuit design is more reminiscent of the classical concept of divisive normalization, which includes averaging of activity levels over multiple neurons (Carandini and Heeger, 2012). Nevertheless, frequency specificity of GABA_B_-mediated adaptation is apparently maintained (Stange et al., 2013).

GABA-mediated control of input strength can also be found in the avian NL circuit: SON shares the input source with NL and is additionally also innervated by NL neurons, creating a differential gain control circuit for the NL (Monsivais and Rubel, 2001; Burger et al., 2005a). Moreover, GABA_B_-receptors are present pre-synaptically on neurons in NL and its excitatory input source, allowing for complex modulation of the ITD circuit depending on the activity level (Burger et al., 2005b; Tang et al., 2009).

In contrast to the avian system, in mammals, the activity-dependent rate adaptations in ILD and ITD coding will ultimately lead to a change in the tuning to the respective parameter, which is a consequence of the specific coding strategy of mammals (**Figure 8**). In the LSO, ILD functions of individual neurons will shift significantly, changing the range of ILDs to which they are sensitive. In contrast, the tuning functions of MSO neurons are not shifted at the single-cell level, as mainly the response gain is modulated. However, these modulations have significant consequences at the level of population coding, particularly in the case of the hemispheric channel model. Since response rates are activity dependent, a lateralized sound-source will generate unequal adaptation in the two hemispheres (with pronounced rate adaptation only in the contralateral channel). This asymmetric change in response rates will therefore shift the intersection of the two tuning channels away from the midline toward the adapting sound-source (**Figure 8C**). Given that the intersection of the two hemispheric channels encodes the perceived (subjective) midline, one would expect that such a lateralized adapting source would shift the perceived location of a subsequently presented sound-source. As noted above, Phillips and Hall (2005), Vigneault-MacLean et al. (2007) first tested this hypothesis in a number of psychophysical paradigms and were able to demonstrate a pronounced shift in the perceived location of sound-sources after prior presentation of a lateralized adapter in human listeners. Our lab has more recently demonstrated that GABA_B_-mediated rate adaptation in the MSO is sufficient to explain these shifts in human perception (Stange et al., 2013).

The primary function of these perceptual shifts seems to be the relative segregation of the adapting and subsequent sound-source, as the reported shifts in location are directed away from the adapter location, i.e., the perceived distance between the sound-sources is increased relative to the actual distance. This interpretation is supported by the finding that the presence of adapting sound-sources increases spatial resolution at the adapter position (Getzmann, 2004).

Taken together, these findings strongly suggest that the binaural system serves to encode the relative separation of concurrent or subsequent sound-sources rather than to provide an absolute representation of position in space.

## CONCLUSION

It is clear from the fossil record that spatial processing of air-borne sound in mammals evolved independently of comparable systems in other vertebrates. Moreover, the electrophysiological circumstances in which such systems emerged were quite diverse, leading sauropsids and frogs into a low-frequency and mammals into a high-frequency world.

Therefore, from the beginning, mammals could make use of ample spectral information for vertical localization using HRTFs, and for lateralization using ILDs.

**FIGURE 9:**
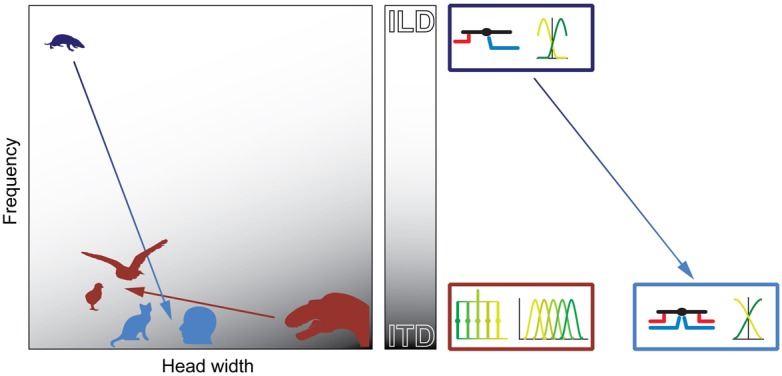
**Physical prerequisites shaped distinct binaural processing and coding strategies in mammals and birds.** Head size (abscissa) and hearing range (ordinate) during the time of the middle-ear development define the binaural cue (gray scale: white = ILD; gray = ITD) that is most easily exploitable for horizontal sound localization by mammals (blue) and archosaurs/birds (brown). The binaural cue in turn shaped the emergence of distinct neuronal mechanisms that are optimized for the processing and encoding of the particular cue (boxes on right-hand side of panel). Early mammals (*Morganucodon,* dark blue) were very small and had high-frequency hearing. Therefore, they used ILDs as original binaural cue. Subsequent evolutionary changes in head size and/or hearing range (e.g., cats or humans, light blue) allowed the use of ITDs. However, the neuronal mechanisms (precise temporal integration of excitatory and inhibitory inputs) and coding principles (population code) remained similar to early, high-frequency hearing mammals (dark and light blue boxes on right-hand side of panel). Early archosaurs (brown) were very large and had low-frequency hearing. Therefore, they used ITDs as original binaural cue. Subsequent evolutionary changes in head size and/or hearing range in birds (e.g., chicks or barn owls, brown) allowed the continued use of ITDs. Thus, the neuronal mechanisms (delay-lines) and coding principles (labeled line) remained the same as in early archosaurs (brown box on right-hand side of panel).

Interaural level differences as the original binaural cue for mammals are encoded via a population code, which largely derives from binaural interactions of excitation and inhibition (**Figure 9**). Later, those mammals that developed low-frequency hearing based their ITD processing, at least partially, on the same neuronal structures (including glycinergic inhibition as an important parameter for tuning ILD/ITD sensitivity in LSO und MSO), similar computations and coding strategies (e.g., a population code at the binaural comparator level, **Figure 9**). This leads to compatible representations of ILDs and ITDs at higher neuronal levels.

In contrast to mammals, archosaurs used ITDs as original binaural cue, because their heads were large and their hearing range was restricted to lower frequencies (**Figure 9**). Birds extended the hearing range only slightly compared to early archosaurs and thus maintained the original neuronal processing mechanisms (based on delay-lines) and coding strategy (**Figure 9**). Thus, physical restrictions shaped different processing mechanisms and coding strategies of binaural cues in birds and mammals.

The mammalian population code is modulated by context even at the binaural detector level. Again, inhibitory inputs (here: GABAergic feedback activating pre-synaptic GABA_B_ receptors) play a major role in adjusting the population output of LSO and MSO. As a consequence, the system serves relative rather than absolute sound localization. This suggests that the main evolutionary constraint in (originally nocturnal) mammals was sound segregation rather than sound localization. More importantly, it suggests a paradigm shift beyond the current understanding of sound localization principles. For several decades the paradigm stated that binaural computations should transform auditory information in a manner similar to how retinotopic processing transforms visual information. In light of the more recent findings of constant remapping in both the auditory (Lee and Middlebrooks, 2011; Maddox et al., 2014) and visual system (Rolfs et al., 2011), we propose instead that the major challenge of localization and orientation is to overcome receptor-surface-bound information encoded via labeled-lines in order to create a flexible representation of external objects in the context of active movement in space and time.

## Conflict of Interest Statement

The authors declare that the research was conducted in the absence of any commercial or financial relationships that could be construed as a potential conflict of interest.
